# Development and validation of a nomogram for circuit lifespan of regional citrate anticoagulation‐continuous renal replacement therapy in intensive care patients with acute kidney injury

**DOI:** 10.1111/nicc.13196

**Published:** 2024-11-07

**Authors:** Zhongbin Chen, Lingai Pan, Jia Zhang, Yanyu Chen, Yi Liu, Ping Jia, Shiya Liu, Bo Wang, Ping Zheng, Feng Chen, Bin Zeng, Wenting Zhang, Qin Yang, Xiaobo Huang, Caixia Xie

**Affiliations:** ^1^ Department of Surgical Intensive Care Unit, Sichuan Provincial People's Hospital, School of Medicine University of Electronic Science and Technology of China Chengdu China; ^2^ Department of Neurosurgery, Sichuan Provincial People's Hospital, School of Medicine University of Electronic Science and Technology of China Chengdu China; ^3^ Department of Emergency Intensive Care Unit, Sichuan Provincial People's Hospital, School of Medicine University of Electronic Science and Technology of China Chengdu China; ^4^ Department of Neurosurgical Intensive Care Unit, Sichuan Provincial People's Hospital, School of Medicine University of Electronic Science and Technology of China Chengdu China; ^5^ School of Medicine University of Electronic Science and Technology of China Chengdu China; ^6^ Department of Oncology, Sichuan Provincial People's Hospital, School of Medicine University of Electronic Science and Technology of China Chengdu China; ^7^ Department of Nursing, Sichuan Provincial People's Hospital, School of Medicine University of Electronic Science and Technology of China Chengdu China

**Keywords:** AKI, circuit lifespan, CRRT, nomogram

## Abstract

**Background:**

Regional citrate anticoagulation‐continuous renal replacement therapy (RCA‐CRRT) has a wide range of applications in clinical practice, but unplanned downtime due to RCA‐CRRT circuit coagulation is as high as 15.75%–66.70%.

**Aim:**

To build a nomogram model for predicting the lifespan circuits of RCA‐CRRT.

**Study design:**

A prospective observational cohort study was conducted in Sichuan, China. The collected clinical data from 404 RCA‐CRRT sessions involving 135 patients were utilized. The patients' basic information, laboratory indicators and RCA‐CRRT parameters were used as independent variables, and the survival status and survival time of RCA‐CRRT circuits were used as dependent variables. A Cox multivariate analysis was performed to build the nomogram model for predicting the lifespan of RCA‐CRRT circuits. The model was validated internally and externally.

**Results:**

The median lifespan of RCA‐CRRT circuits was 28.0 (12.0–46.5) h, and the unplanned downtime rate was 23.76%. In the Cox multivariate analysis, venous pressure, haemoglobin, Sequential Organ Failure Assessment (SOFA), lactate, and blood transfusion were identified as statistically significant predictive factors for the lifespan of RCA‐CRRT circuits (*p* < .05). Subsequently, a nomogram model for predicting the lifespan of RCA‐CRRT circuits was developed. The AUC values for internal and external validation within the 12–72‐h timeframe ranged from 0.648 to 0.816 and 0.613 to 0.956, respectively. Both the calibration curve and clinical decision curve demonstrated the model's good performance.

**Conclusion:**

The nomogram model developed in this study demonstrates its efficacy in accurately predicting the lifespan circuits of RCA‐CRRT.

**Relevance to Clinical Practice:**

Clinical nurses can use the prediction model to assess the lifespan of RCA‐CRRT circuits, so as to formulate a personalized RCA‐CRRT treatment plan for patients, thus reducing the unplanned downtime of RCA‐CRRT.

**What is known about the topic**

- In clinical practice, CRRT nurses cannot predict the lifespan of CRRT circuit in advance and cannot formulate individualized treatment plans, which is easy to cause unplanned CRRT shutdown, loss of blood pressure of patients and failure to achieve the treatment goal.

**What this paper adds**

- When using regional citrate anticoagulation for CRRT, special attention should be paid to the venous pressure of the CRRT circuit, as it is a crucial factor influencing the lifespan of the CRRT circuit.
- Nurses operating CRRT should not only focus on the pressure of the circuit, but also evaluate the internal environment of patients (haemoglobin, lactate, SOFA score and blood transfusion).
- CRRT nurses can use the RCA‐CRRT prediction model to develop individualized treatment plans and reduce the risk of unplanned downtime.

## 1 INTRODUCTION

The circuit lifespan of RCA‐CRRT refers to the duration of dialysis circuit usage during continuous renal replacement therapy with regional citrate anticoagulation.^1^, ^2^ Currently, the circuit lifespan of continuous renal replacement therapy serves as a crucial metric for assessing the quality of CRRT in clinical practice. International studies indicate that the RCA‐CRRT circuit lifetime is 50–71.1 h,^3^, ^4^ while domestic studies in China report a shorter range of 12–48 h.^5^, ^6^, ^7^ This suggests that the RCA‐CRRT circuit lifespan in China is relatively short.

The relatively short RCA‐CRRT circuit lifetime leads to unplanned downtime due to factors such as disease treatment (e.g., death, transfer out of the intensive care unit, and examinations) and circuit coagulation.^8^ Circuit coagulation is the main cause of unplanned downtime in RCA‐CRRT.^9^ Research indicates that the rate of unplanned downtime due to RCA‐CRRT circuit coagulation is as high as 15.75%–66.7%,^6^, ^10^, ^11^ which results in a series of problems.^12^ These include challenges such as the inability of the dialysis circuit to return 200–500 mL of blood to the patient's body, resulting in blood loss; an increase in the number of upper and lower machine times for patients, thereby elevating the risk of iatrogenic infection; the need for repeated CRRT, adding to the workload of medical staff; and an upsurge in the use of dialysis circuits, consequently raising medical consumables costs for patients. It is evident that the shortened lifespan of RCA‐CRRT poses significant risks to patients and imposes a substantial medical burden. Identifying the influencing factors of RCA‐CRRT circuit lifespan, developing prediction tools for RCA‐CRRT circuit lifespan, and formulating personalized medical care decisions based on these tools can enhance the expected compliance rate of RCA‐CRRT circuit lifespan and mitigate the harm caused by circuit coagulation.^2^

The prediction model for CRRT circuit coagulation has been developed by Fu et al., specifically focusing on the circuit's survival status within the initial 24 h.^13^ However, this model lacks the capability to predict the circuit's survival beyond the initial 24‐h period. On the other hand, a CRRT circuit coagulation prediction model with an AUC prediction performance of 0.865 has been constructed, but this model is designed to predict the survival status of circuits under various anticoagulation methods for CRRT (such as low molecular weight heparin, citrate anticoagulation, no heparin anticoagulation, and argatroban anticoagulation).^14^ Unfortunately, this model does not specifically address the prediction of RCA‐CRRT circuit lifespan. In a related study, Tang et al. have studied the influencing factors of RCA‐CRRT circuit lifespan, although a dedicated prediction model for this purpose has not been constructed.^15^ As a result, there is currently a gap in research regarding the development of prediction models specifically tailored to forecast RCA‐CRRT circuit lifespan. Citrate anticoagulation has been recommended as the preferred anticoagulation method for CRRT by the KDIGO guidelines^16^ and RCA‐CRRT has a wide range of applications in clinical practice. Therefore, it is necessary to conduct research on constructing prediction models for RCA‐CRRT circuit lifespan.

## 2 AIM OF THE STUDY

The aim of this study is to explore the factors that influence the lifespan of RCA‐CRRT circuits using a prospective observational research method and to construct a nomogram prediction model for RCA‐CRRT circuit lifespan. This model can be used to develop individualized RCA‐CRRT treatment plans for patients, providing practical evidence to reduce the harm caused by shortened circuit lifespan due to circuit coagulation.

## 3 METHOD

### 3.1 Study design and study sample

This was a prospective, observational study conducted at a single centre in the surgical intensive care unit of a hospital in western China, spanning from December 2021 to November 2022.

Inclusion criteria required participants to meet all three of the following conditions: ① age > 18 years old; ② diagnosis of acute kidney injury (AKI); and ③ necessity for RCA‐CRRT treatment. Exclusion criteria applied if any of the following conditions were met: ① pregnancy in patients; ② severe dysfunctionality issues with central venous dialysis catheter outlet; and ③ abnormal parathyroid function. Censorship criteria led to exclusion if any of the following conditions occurred: ① missing data >5%; ② termination of RCA‐CRRT due to patient agitation; ③ termination of RCA‐CRRT due to equipment failure during treatment; and ④patient death during RCA‐CRRT treatment.

Based on the 10EPV principle,^17^ the preliminary study of this research indicates an approximate 30% incidence rate of outcome events. Tang's study on factors influencing the circuit lifespan of RCA‐CRRT incorporates eight independent variables.^15^ Consequently, the minimum sample size required for the training set is calculated as 8 × 10÷30%, equalling 267 sessions. Given a training set to validation set division ratio of 8:2, the minimum necessary sample size for the validation set amounts to 67 cases. Notably, the actual sample sizes included in the training set and validation set are 312 sessions and 72 sessions, respectively, exceeding the minimum requirements of 267 sessions and 67 sessions.

### 3.2 CRRT prescription

The research centre employs Fresenius dialysis machines, utilizing AV1000S filters. Dialysis is conducted with calcium‐containing replacement fluid from Qingshan Lifkang Co., Ltd., following a prescribed dosage of 25–30 mL/kg/h. The chosen treatment mode is continuous venovenous haemodialysis (CVVHD). Post‐filter ionized calcium levels are maintained between 0.2 and 0.4 mmol/L, while free calcium ion concentration ranges from 1.0 to 1.2 mmol/L. The blood flow velocity of the dialysis circuit stands at 120 mL/min. Initial doses of 4% sodium citrate are set at 180 mL/h, adjusted dynamically based on post‐filter ionized calcium levels. Calcium gluconate is used as the calcium agent for injection, with an initial dose of 10 mL/h. This dose is also adjusted dynamically based on the calcium ion concentration. The targeted maintenance range for serum sodium is 136–145 mmol/L, while the pre‐set concentration of sodium ions in the ready‐to‐use dialysate is 141 mmol/L. In cases where the blood sodium level deviates from this range, either too low or too high, adjustments can be made to the sodium infusion or tube feeding, as prescribed by the physician.

### 3.3 Diagnosis of RCA‐CRRT circuit lifespan

In this study, the circuit lifespan of RCA‐CRRT refers to the duration of the dialysis circuit's usage during the RCA‐CRRT process, with the circuit survival status at the end of RCA‐CRRT as the outcome indicator. The survival status includes normal downtime after achieving the target treatment volume and unplanned downtime due to blood coagulation in the circuit before achieving the treatment goal. The diagnostic criteria for circuit coagulation include transmembrane pressure > 250 mmHg (1 mmHg = 0.133 kPa), venous pressure > 350 mmHg, filter coagulation grade II or higher, and various alarms that cannot be excluded as leading to disconnection.^18^ At the end of RCA‐CRRT treatment, two researchers simultaneously assess the circuit's status.

### 3.4 Screening of independent variables

Twenty‐four independent variables were included in the study by reviewing the research on CRRT circuit lifespan and advices from experts working in CRRT, including red blood cells, haemoglobin, platelets, lactic acid, pH, C‐reactive protein, PCT, D‐dimer, PT, APTT, haematocrit, BMI, transfusion of red blood cell suspension (yes/no), infusion of fat emulsion (yes/no), plasma infusion (yes/no), platelet infusion (yes/no), administration of anticoagulants (yes/no), circuit blood flow velocity, venous pressure (VP), transmembrane pressure (TMP), ultrafiltration rate, treatment mode, post‐membrane calcium ion concentration and catheter site.

### 3.5 Data collection

At the start of RCA‐CRRT, patients' general information (age, gender, Body Mass Index (BMI), hospitalization number, diagnosis, underlying diseases, medication history) and SOFA scores were collected through the hospital's electronic medical record system. Within 12 h, laboratory indicators (liver function, renal function, blood routine, biochemical calcium, coagulation function, PH value, haematocrit) were also collected. During the RCA‐CRRT process, treatment parameter data were collected, including treatment mode, central venous dialysis catheter site, blood flow rate, venous pressure, transmembrane pressure, systemic calcium ion concentration, post‐membrane calcium ion concentration and blood flow through the circuit. At the end of RCA‐CRRT, the reasons for termination and circuit coagulation ratings were collected.

### 3.6 Statistical analysis

Statistical analysis was performed using SPSS 24.0 software. Count data were described using frequency counts, and intergroup comparisons were performed using the chi‐square test or Fisher's exact test. Measurement data were described using means ± standard deviations, medians or interquartile ranges, and intergroup comparisons were performed using the *t*‐test. *p* values were bilateral tests, and a *p* value of less than .05 was considered statistically significant. LASSO regression analysis and Cox regression analysis were performed using R4.1.0 software to build the RCA‐CRRT circuit life prediction model. Survival curves, ROC curves, calibration curves and decision curves were plotted. In place of missing values, we employ central tendency metrics, including the mean, median and mode. If the variable data follow a normal distribution, we utilize the mean value to address missing data. Conversely, when the variable data exhibit a non‐normal distribution, we employ the median value. For qualitative variables, we utilize the mode to address missing data.

The construction of the RCA‐CRRT circuit lifespan prediction model was divided into two steps. In the first step, the LASSO regression model was used to screen for characteristic variables of RCA‐CRRT circuit lifespan. In the second step, the Cox regression model was used to analyse the characteristic variables of RCA‐CRRT circuit life and construct a nomogram model for RCA‐CRRT circuit lifespan.

In this study, the data were divided into training sets and validation sets. The training set was used for internal validation, and the validation set was used for external validation. The following three indicators were used to evaluate the model: area under the receiver operating characteristic (ROC) curve (AUC), calibration curve (Hosmer–Lemeshow test, HL), and decision curve analysis (DCA).

## 4 ETHICAL AND RESEARCH APPROVALS

This study has been approved by Sichuan Provincial People's Hospital ethics committee, with the project number: ethics (research) 2022 No. 97. This study has been reviewed and registered at the China Clinical Trial Registry, with the registration number: ChiCTR2200065116.

## 5 RESULTS

### 5.1 Clinical characteristics of RCA‐CRRT

A total of 135 patients underwent 438 RCA‐CRRT sessions, of which 34 sessions were excluded, and 404 sessions were ultimately included in this study. The median RCA‐CRRT circuit life was 28 (12–46.5) h, with a total treatment time of 13 029 h. There were 308 planned downtimes, accounting for 76.24%, and 96 unplanned downtimes due to circuit coagulation, with an unplanned downtime rate of 23.76%. Please see Supplement 1 for details.

Among the 404 RCA‐CRRT sessions, the first 312 sessions were used as the training set, and the next 92 sessions were used as the validation set. Univariate analysis was used to analyse the influencing factors between the two groups, and it was found that there were no significant differences in influencing factors between the two groups (Table 1).

**TABLE 1:** Baseline data for training and validation sets.

Variable	Total (*n* = 404)	Training set (*n* = 312)	Validation set (*n* = 92)	*p*‐value
Unplanned downtime rate, s (%)	96 (23.76%)	78 (25%)	18 (19.57%)	.262
Circuit lifespan^a^ (h, median, IQR)	32.6 (20.3)	32.3 (20.1)	33.7 (20.9)	.575
BMI				.52
<18.5	81 (20.0%)	60 (19.2%)	21 (22.8%)	
18.5–23.9	210 (52.0%)	161 (51.6%)	49 (53.3%)	
24–27.9	87 (21.5%)	72 (23.1%)	15 (16.3%)	
>27.9	26 (6.44%)	19 (6.09%)	7 (7.61%)	
Location				.97
Right femoral	222 (55.0%)	170 (54.5%)	52 (56.5%)	
Left femoral	102 (25.2%)	80 (25.6%)	22 (23.9%)	
Right jugular	59 (14.6%)	45 (14.4%)	14 (15.2%)	
Left jugular	21 (5.20%)	17 (5.45%)	4 (4.35%)	
Hb				1
<115 g/L	398 (98.5%)	307 (98.4%)	91 (98.9%)	
115–150 g/L	4 (0.99%)	3 (0.96%)	1 (1.09%)	
>150 g/L	2 (0.50%)	2 (0.64%)	0 (0.00%)	
RBC				.738
<3.80 ´ 10^12^/L	396 (98.0%)	306 (98.1%)	90 (97.8%)	
3.80–5.10 ´ 10^12^/L	7 (1.73%)	5 (1.60%)	2 (2.17%)	
>5.10 ´ 10^12^/L	1 (0.25%)	1 (0.32%)	0 (0.00%)	
PLT				.425
<101 ´ 10^9^/L	250 (61.9%)	197 (63.1%)	53 (57.6%)	
101–320 ´ 10^9^/L	142 (35.1%)	107 (34.3%)	35 (38.0%)	
>320 ´ 10^9^/L	12 (2.97%)	8 (2.56%)	4 (4.35%)	
PT				.3
11–17 s	300 (74.3%)	236 (75.6%)	64 (69.6%)	
>17 s	104 (25.7%)	76 (24.4%)	28 (30.4%)	
APTT				.656
25–47 s	357 (88.4%)	274 (87.8%)	83 (90.2%)	25–47 s
>47 s	47 (11.6%)	38 (12.2%)	9 (9.78%)	
D‐Dimer				.661
0–0.5 mg/L	7 (1.73%)	5 (1.60%)	2 (2.17%)	
>0.5 mg/L	397 (98.3%	307 (98.4%)	90 (97.8%)	
HCT				0.425
<35%	355 (87.9%)	276 (88.5%)	79 (85.9%)	
35–45%	45 (11.1%)	32 (10.3%)	13 (14.1%)	
>45%	4 (0.99%)	4 (1.28%)	0 (0.00%)	
INR				.699
0.8–1.5	294 (72.8%)	229 (73.4%)	65 (70.7%)	
>1.5	110 (27.2%)	83 (26.6%)	27 (29.3%)	
CRP				.542
0–8 mg/L	16 (3.96%)	14 (4.49%)	2 (2.17%)	
>8 mg/L	388 (96.0%	298 (95.5%)	90 (97.8%)	
PCT				.702
0–0.5 ng/mL	10 (2.48%)	7 (2.24%)	3 (3.26%)	
>0.5 ng/mL	394 (97.5%)	305 (97.8%)	89 (96.7%)	
BFR (mL/min, mean, SD)	121 (9.36)	121 (9.40)	122 (9.20)	.25
Lac (mmol/L, median, IQR)	2.84 (2.72)	2.81 (2.72)	2.94 (2.73)	.69
RBC infusion				.307
No	223 (55.2%)	177 (56.7%)	46 (50.0%)	
Yes	181 (44.8%)	135 (43.3%)	46 (50.0%)	
PLT infusion				.232
No	343 (84.9%)	269 (86.2%)	74 (80.4%)	
Yes	61 (15.1%)	43 (13.8%)	18 (19.6%)	
Plasma infusion				1
No	331 (81.9%)	256 (82.1%)	75 (81.5%)	
Yes	73 (18.1%)	56 (17.9%)	17 (18.5%)	
Albumin infusion				.993
No	178 (44.1%)	138 (44.2%)	40 (43.5%)	
Yes	226 (55.9%)	174 (55.8%)	52 (56.5%)	
FE infusion				.917
No	119 (29.5%)	91 (29.2%)	28 (30.4%)	
Yes	285 (70.5%)	221 (70.8%)	64 (69.6%)	
Anticoagulant infusion				1
No	309 (76.5%)	239 (76.6%)	70 (76.1%)	
Yes	95 (23.5%)	73 (23.4%)	22 (23.9%)	
VP (mean, SD)	45.3 (16.9)	44.8 (16.5)	46.9 (18.2)	.318
TMP^a^ (median, IQR)	37.1 (26.8)	36.0 (26.1)	41.0 (28.8)	.141
P‐Ca				.99
0.20–0.40	126 (31.2%)	97 (31.1%)	29 (31.5%)	
0.40–0.45	84 (20.8%)	66 (21.2%)	18 (19.6%)	
0.46–0.50	91 (22.5%)	70 (22.4%)	21 (22.8%)	.46–.50
>0.50	103 (25.5%)	79 (25.3%)	24 (26.1%)	
PH				.597
<7.35	141 (34.9%)	107 (34.3%)	34 (37.0%)	
7.35–7.45	185 (45.8%)	147 (47.1%)	38 (41.3%)	
>7.45	78 (19.3%)	58 (18.6%)	20 (21.7%)	
SOFA score (mean, SD)	11.5 (4.83)	11.5 (4.78)	11.7 (5.03)	.658

Abbreviations: APTT, activated partial thromboplastin time; BFR, blood flow rate; BMI, body mass index; CRP, C‐reactive protein; FE, fat emulsion; Hb, haemoglobin; HCT, haematocrit; INR, international normalized ratio; Lac, lactic acid; P‐Ca, post‐membrane calcium ion concentration; PCT, procalcitonin; PH, potential of hydrogen; PLT, blood platelet count; PT, prothrombin time; RBC, red blood cell; SOFA, sequential organ failure assessment; TMP, transmembrane pressure; VP, venous pressure.

^a^ Circuit lifespan, TMP using Wilcoxon rank sum test.

### 5.2 Univariate analysis results

This study focused on the survival status and survival time of RCA‐CRRT circuits as outcome variables. COX univariate analysis is shown in Table 2, and eight variables were found to have statistically significant differences: Lac (*p* = .042), VP (*p* < .001), TMP (*p* = .006), BMI (*p* = .016), Hb (*p* < .001), RBC (*p* < .001), blood transfusion (*p* < .001) and SOFA score (*p* = .005). These results are detailed in Table 2.

**TABLE 2:** Univariate analysis of RCA‐CRRT circuit life.

Variable	β	SE	Wald	*p*	HR	95.0 %CI	
						Lower limit	Upper limit
BFR	0.015	0.011	1.87	.171	1.015	0.993	1.038
Lac	−0.122	0.060	4.128	.042	0.885	0.787	0.996
VP	0.030	0.006	24.292	<.001	1.031	1.019	1.043
TMP	0.012	0.004	7.541	.006	1.012	1.003	1.021
SOFA	0.072	0.026	7.804	.005	1.075	1.022	1.131
BMI (vs. 18.5–23.9)			10.393	.016			
<18.5	−0.195	0.259	0.569	.451	0.823	0.496	1.366
24–27.9	0.977	0.506	3.735	.053	2.656	0.986	7.155
>27.9	−0.887	0.432	4.215	.04	0.412	0.177	0.961
Location (vs. Right femoral)			0.987	.804			
Left femoral	0.336	0.600	0.313	.576	1.399	0.432	4.536
Right jugular	0.173	0.618	0.078	.78	1.189	0.354	3.992
Left jugular	0.070	0.647	0.012	.913	1.073	0.302	3.812
Hb (vs. 115–150 g/L)			31.211	<.001			
<115 g/L	−2.696	1.056	6.518	.011	0.067	0.009	0.535
>150 g/L	−4.182	0.796	27.599	<.001	0.015	0.003	0.073
RBC (vs. 3.80–5.10 10^12^/L)	−2.12	0.52	16.656	<.001	0.12	0.040	0.330
PT (vs. 11–17 s)	−0.067	0.252	0.071	.79	0.935	0.570	1.533
APTT (vs. 25–47 s)	−0.168	0.325	0.267	.606	0.845	0.447	1.600
D‐Dimer (vs. 0–0.5 mg/L)	−0.409	1.007	0.165	.685	0.664	0.092	4.785
HCT (vs. 35–45%)			0.977	.614			
<35%	−0.613	0.776	0.624	.429	0.542	0.118	2.478
>45%	−0.698	0.720	0.941	.332	0.497	0.121	2.038
INR (vs. 0.8–1.5)	0.165	0.252	0.425	.514	1.179	0.719	1.933
CRP (vs. 0–8 mg/L)	−0.190	0.717	0.070	.791	0.827	0.203	3.374
PCT (vs. 0–0.5 ng/mL)	0.438	0.717	0.372	.542	1.549	0.38	6.321
RBC infusion (vs. No)	0.891	0.241	13.679	<.001	2.438	1.52	3.911
Plasma infusion (vs. No)	0.507	0.298	2.897	.089	1.661	0.926	2.978
Albumin infusion (vs. No)	0.456	0.237	3.703	.054	1.578	0.992	2.510
FE infusion (vs. No)	0.215	0.249	0.744	.388	1.239	0.761	2.018
PLT infusion (vs. No)	0.191	0.306	0.392	.531	1.211	0.665	2.205
Anticoagulant infusion (vs. No)	0.453	0.314	2.082	.149	1.573	0.850	2.911
P‐Ca (vs. 0.2–0.4)			0.154	.985			
0.4–0.45	−0.033	0.303	0.012	.913	0.967	0.535	1.75
0.46–0.5	−0.120	0.345	0.121	.728	0.887	0.451	1.745
>0.5	0.002	0.313	0	.994	1.002	0.543	1.850
PH (vs. 7.35–7.45)			0.618	.734			
<7.35	0.271	0.357	0.577	.447	1.311	0.652	2.639
>7.45	0.260	0.364	0.509	.475	1.297	0.635	2.648
PLT (vs. 101–320 ´ 10^9^/L)			0.151	.927			
<101 ´ 10^9^/L	−0.259	0.742	0.122	.727	0.772	0.180	3.302
>320 ´ 10^9^/L	−0.194	0.721	0.072	.788	0.824	0.201	3.383

Abbreviations: APTT, activated partial thromboplastin time; BFR, blood flow rate; BMI, body mass index; CRP, C‐reactive protein; FE, fat emulsion; Hb, haemoglobin; HCT, haematocrit; INR, international normalized ratio; Lac, lactic acid; P‐Ca, post‐membrane calcium ion concentration; PCT, Procalcitonin; PH, potential of hydrogen; PLT, blood platelet count; PT, prothrombin time; RBC, red blood cell; SOFA, sequential organ failure assessment; TMP, transmembrane pressure; VP, venous pressure.

### 5.3 Lasso regression analysis

In this study, the Lasso regression analysis method was used to screen the characteristic variables influencing the RCA‐CRRT circuit lifespan (Supplement 2), which were haemoglobin, venous pressure, SOFA score, lactate and blood transfusion. Finally, the five selected characteristic variables were used as predictors in the RCA‐CRRT circuit lifespan prediction model.

### 5.4 Multivariable analysis results

In the training set (*n* = 312), five predictive factors, including haemoglobin, venous pressure, SOFA score, lactate and blood transfusion, were used as predictors. Through multivariate analysis, it was found that all five predictive factors were statistically significant. A prediction model for the RCA‐CRRT circuit lifespan was constructed, detailed in Table 3.

**TABLE 3:** Multivariable analysis of RCA‐CRRT circuit lifespan.

Variable	β	SE	Wald	*p*	HR	95.0%CI	
						Lower limit	Upper limit
Hb (vs. Normal)			15.687	<.001			
Low	0.07	0.61	0.013	.909	1.072	0.324	3.544
High	1.296	0.651	3.96	.047	3.655	1.02	13.103
VP (10 mmHg)	0.031	0.008	16.809	<.001	10.32	10.16	10.47
SOFA	0.096	0.033	8.586	.003	1.1	1.032	1.173
Lac	−0.178	0.096	3.443	.026	0.86	0.75	0.98
RBC infusion (vs. No)	−0.655	0.29	5.087	.024	0.519	0.294	0.918

Abbreviations: Hb, haemoglobin; Lac, lactic acid; RBC, red blood cell; SOFA, sequential organ failure assessment; VP, venous pressure.

### 5.5 Development of a nomogram on the RCA‐CRRT circuit lifespan

**FIGURE 1:**
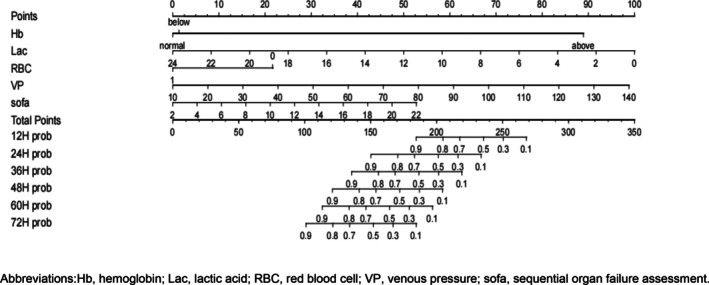
Nomogram on the regional citrate anticoagulation‐continuous renal replacement therapy circuit lifespan.

The nomogram of RCA‐CRRT circuit life prediction model is constructed using R 4.1.0 software, as shown in Figure 1. The nomogram is used as follows: The scores for each predictor variable are summed to obtain a total score. A vertical line is drawn from the total score on the score axis, resulting in survival probabilities at various time intervals.

### 5.6 Validation and evaluation of a nomogram on the RCA‐CRRT circuit lifespan in the training set

The model was internally validated in the training set. The AUC values at 12, 24, 36, 48, 60 and 72 h were 0.816, 0.786, 0.759, 0.744, 0.705 and 0.648, respectively, indicating good predictive performance of the model (Supplement 3). The calibration curve and standard curve of the model fit well at 12, 24, 36 and 48 h but were only basically consistent at 60 and 72 h, indicating that the model's prediction of RCA‐CRRT circuit life is consistent with actual clinical outcomes (Supplement 4). The clinical decision curve of the model at various time points shows that the net benefit obtained from predicting survival probability within a certain range is relatively high and has good clinical validity, as shown in Supplement 5.

### 5.7 Validation and evaluation of a nomogram on RCA‐CRRT circuit lifespan in the validation set

The model was externally validated in the validation set (*n* = 92). The AUC values at 12, 24, 36, 48, 60 and 72 h were 0.956, 0.795, 0.686, 0.771, 0.755 and 0.613, respectively, indicating good predictive performance of the model at various time intervals of RCA‐CRRT circuit life. Supplement 6 shows the detailed information. The calibration curve and standard curve of the model fit well, further confirming that the model's prediction of RCA‐CRRT circuit lifespan is consistent with actual clinical outcomes. The detailed information is shown in Supplement 7. The clinical decision curve of the model in the validation set shows that the net benefit obtained from predicting survival probability within a certain range is relatively high (Supplement 8), further demonstrating the good clinical validity of the model.

## 6 DISCUSSION

### 6.1 RCA‐CRRT circuit lifespan

This study included 135 patients and collected clinical data on 404 cases of RCA‐CRRT. The median lifespan of the circuit was 28.0 (12.0–46.5) h. Unplanned downtime due to circuit clotting occurred in 96 cases, with an unplanned downtime rate of 23.76%. Compared with other research centres' RCA‐CRRT, Cassina's research centre had an unplanned downtime rate of 33.33%,^19^ and Harin Rhee's research centre had an unplanned downtime rate of 22.2%.^3^ The possible reasons are as follows: first, the treatment protocols of RCA‐CRRT in this research centre are unified, such as dialysate/replacement fluid volume of 25–30 mL/kg/h and blood flow rate of 120 mL/min; second, this research centre has a separate CRRT medical care management team, which improves the supervision quality of RCA‐CRRT, such as standardized online, offline and alarm handling.

### 6.2 Factors affecting the circuit lifespan of RCA‐CRRT

Venous pressure is an important influencing factor of RCA‐CRRT circuit lifespan. It is a risk predictor of RCA‐CRRT circuit lifespan (HR = 10.28, *p* < .01). In this study, the first 12 h' mean venous pressure was selected as the predictive variable for the model through the quartile method. This finding is consistent with a study by Li et al.^20^ Citrate has the advantages of good biocompatibility, good anticoagulation effect and a stronger protective effect on filters.^21^ In RCA‐CRRT, circuit coagulation mostly occurs in the venous chamber, which is characterized by increased venous pressure.^22^ Moreover, in this study, the blood flow rate was 120 mL/h, excluding the impact of different blood flow rates on venous pressure.^6^ Therefore, it can be seen that the venous pressure value in this study reflects the generation of blood clots in the venous chamber. Therefore, health care professionals should attentively monitor dynamic changes in venous pressure when connecting RCA‐CRRT to the circuit. Timely assessment of blood clots in the venous chamber, identification of problems, determination of causes and prompt intervention are crucial aspects of managing the circuit effectively.

Haemoglobin is a risk predictor of RCA‐CRRT circuit lifespan (HR = 3.66, *p* = .047), which is consistent with the study results of Eduardo de Oliveira Valle et al.^23^ When haemoglobin increases, the blood of patients becomes more concentrated and viscous. Therefore, for patients with elevated haemoglobin undergoing RCA‐CRRT, treatment modes that can dilute the blood viscosity of the circuit should be selected (such as CVVH or CVVHDF) to prolong the circuit lifespan.^24^

Lactate is a protective factor for RCA‐CRRT circuit lifespan (HR = 0.86, *p* = .026). This study result is consistent with the results of others on the risk prediction model of CRRT circuit coagulation.^13^, ^25^ When lactate increases, the liver synthesis of coagulation factors will be inhibited, reducing coagulation factors in the body and reducing the risk of CRRT circuit coagulation. However, lactate is >4.0 mmol/L: it is a relative contraindication for CRRT to use citrate anticoagulation.^26^ Therefore, before RCA‐CRRT treatment, clinical medical staff should pay attention to patients' lactate values.

The SOFA score is a risk predictor of RCA‐CRRT circuit lifespan (HR = 1.1, *p* = .001). The higher the SOFA score before treatment, the more organ failures in patients, which can lead to disorders in the patient's internal environment: a disorder of acid–base balance, insufficient tissue perfusion and hypoxemia.^26^, ^27^ This can inhibit the chelation of citrate and calcium ions in the CRRT circuit, thereby affecting the anticoagulation effect of the CRRT circuit. Therefore, before RCA‐CRRT treatment, clinical medical staff should check the patient's SOFA score to prepare for predicting the lifespan of the RCA‐CRRT circuit.

### 6.3 The nomogram for RCA‐CRRT circuit lifespan

In this study, five predictors were selected through Lasso regression and COX regression, including haemoglobin, venous pressure, lactate, SOFA score and blood transfusion, to construct a nomogram prediction model for the lifespan of RCA‐CRRT circuits. Internal validation showed that the AUC values at 12, 24, 36, 48, 60 and 72 h were 0.816, 0.786, 0.759, 0.744, 0.705 and 0.648, respectively. External validation revealed AUC values of 0.956, 0.795, 0.686, 0.771, 0.755 and 0.613 at the same time points, indicating good predictive performance of the model from 12 to 60 h but poorer performance at 72 h. To further assess the model's predictive capability, we applied it to both the training and validation sets and re‐predicted the samples. Risk levels above 50% were defined as high‐risk groups, while those below 50% were categorized as low‐risk groups based on model predictions. Survival curves were used to analyse the survival status of the two groups. The results showed that the survival rate was higher in the low‐risk group than in the high‐risk group (*p* < .05), as shown in Supplements 9 and 10. Overall, the RCA‐CRRT circuit lifespan prediction model constructed in this study demonstrates good predictive performance and can effectively identify the survival status of RCA‐CRRT circuits. Clinicians can utilize this nomogram model to predict the survival status of RCA‐CRRT circuits at various time points and to develop individualized treatment plans for patients to improve the quality of CRRT treatment.

## 7 LIMITATIONS

The study has several limitations. First, the external validation of the model revealed AUC values at the 36 and 72 h time points of 0.686 and 0.613, respectively, suggesting a need for further improvement in predictive performance. Future researchers may consider optimizing predictive factors to enhance the model's accuracy, such as incorporating the variable of post‐membrane calcium ion concentration using artificial intelligence methods. This could address the problem of continuous changes over time in post‐membrane calcium ion concentration, making it more feasible for research inclusion. Second, although clinicians can use the RCA‐CRRT circuit lifespan prediction model constructed in this study to quickly predict the survival probability of RCA‐CRRT circuits at various time points, they cannot obtain a specific threshold for circuit lifespan. Subsequent studies could explore ongoing RCA‐CRRT with circuit coagulation as the treatment endpoint, constructing a time series prediction model focused solely on time as the outcome measure. This approach might offer clinicians precise thresholds for RCA‐CRRT circuit lifespan, enhancing the clinical application value of the model. Finally, this research is a single‐center study. Future research efforts should include multi‐center external validation methods to validate the predictive performance of the model across diverse clinical settings.

## 8 IMPLICATIONS FOR PRACTICE

Clinical nurses can use the model to accurately assess the lifespan of RCA‐CRRT circuits, thereby facilitating the optimization of CRRT treatment plans. This approach significantly minimizes the risk of unscheduled RCA‐CRRT discontinuation and effectively mitigates the range of adverse consequences (such as repeated blood transfusion, repeated access to the machine, etc.) associated with such unexpected interruptions.

## FUNDING INFORMATION

This work was supported by “Health Science Research Project of Sichuan Province (Chuan gan Yan 2024‐230)”, “Health Science Research Project of Sichuan Province (Chuan gan Yan 2023‐223)”, “SichuanScience and Technology Program (2023YFS0067)”, “China Postdoctoral Science Foundation (2022M720656)”.

## CONFLICT OF INTEREST STATEMENT

The authors declare that they have no known competing financial interests or personal relationships that could have appeared to influence the work reported in this paper.

## Supporting information


**Data S1.** Supporting information.

**Data S2.** Supporting information.

**Data S3.** Supporting information.

**Data S4.** Supporting information.

**Data S5.** Supporting information.

**Data S6.** Supporting information.

**Data S7.** Supporting information.

**Data S8.** Supporting information.

**Data S9.** Supporting information.

**Data S10.** Supporting information.

## Data Availability

The data that support the findings of this study are available from the corresponding author upon reasonable request.
