# Supplementary figures and images for: Development and validation of a nomogram for circuit lifespan of regional citrate anticoagulation‐continuous renal replacement therapy in intensive care patients with acute kidney injury
Source: Nurs Crit Care. 2024 Nov 7;30(4):e13196. doi: 10.1111/nicc.13196 (PMC12208818; doi:10.1111/nicc.13196)

## Supplement 1. enrollment and outcomes

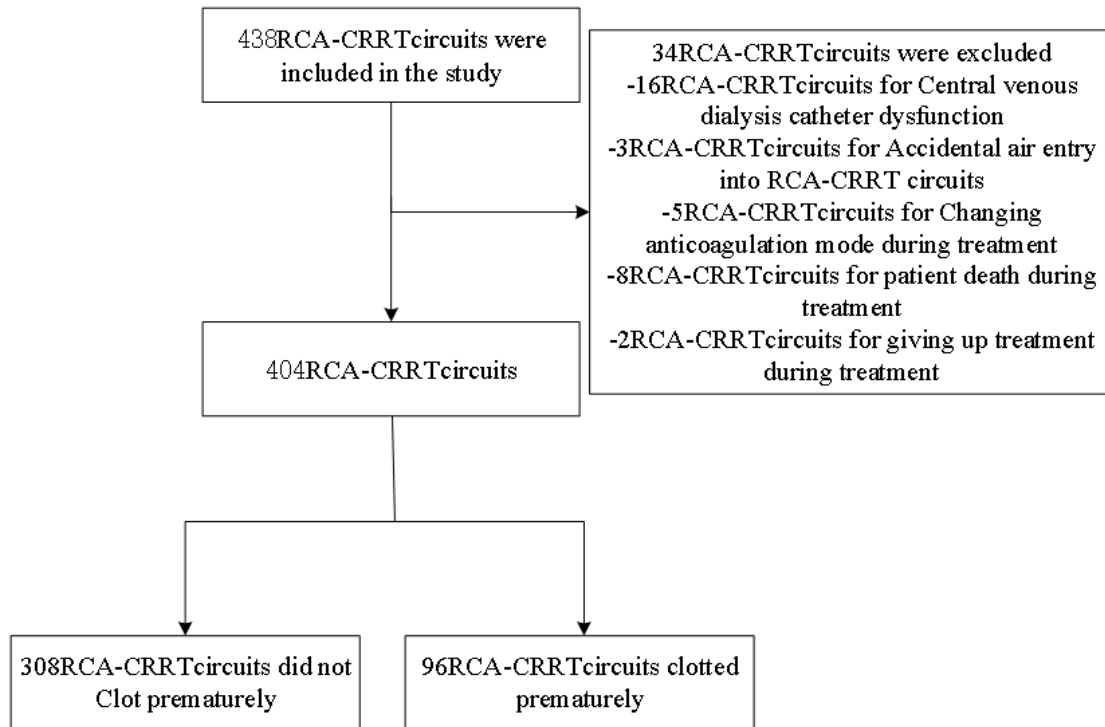

Supplement: Supplementary file 1 — Data S1. Supporting information. [file NICC-30-0-s008.pdf]

Supplement 3.ROC curve of the training set for the RCA-CRRT circuit  
lifespan prediction model

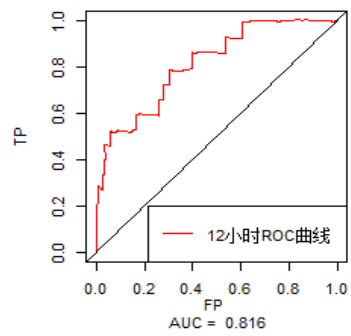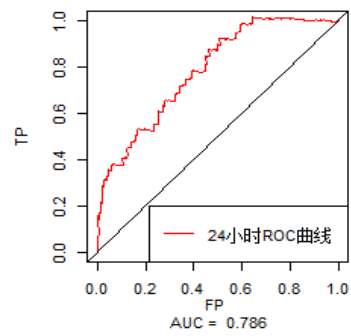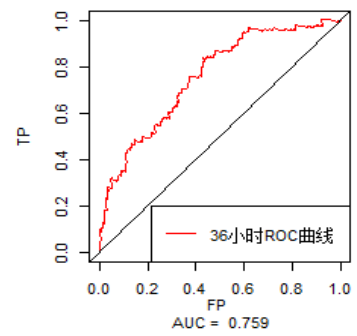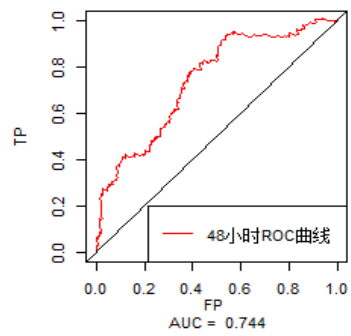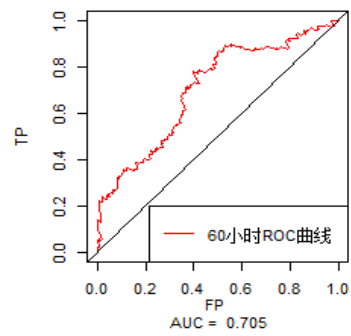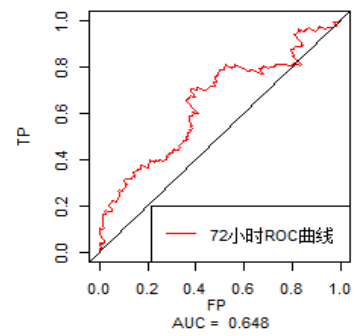

Supplement: Supplementary file 3 — Data S3. Supporting information. [file NICC-30-0-s002.pdf]

## Supplement 6. ROC curve of the validation set for the RCA-CRRT circuit

### lifespan prediction model

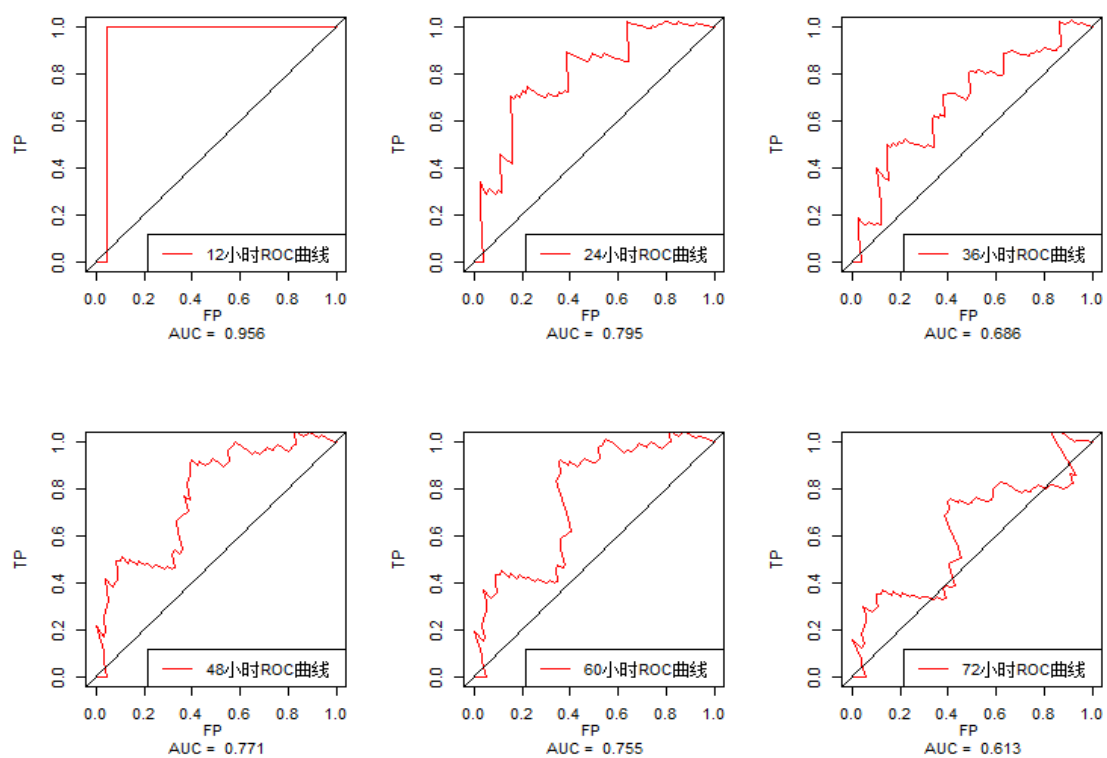

Supplement: Supplementary file 6 — Data S6. Supporting information. [file NICC-30-0-s005.pdf]

Supplement 9.Survival curves of high and low risk groups in the training set

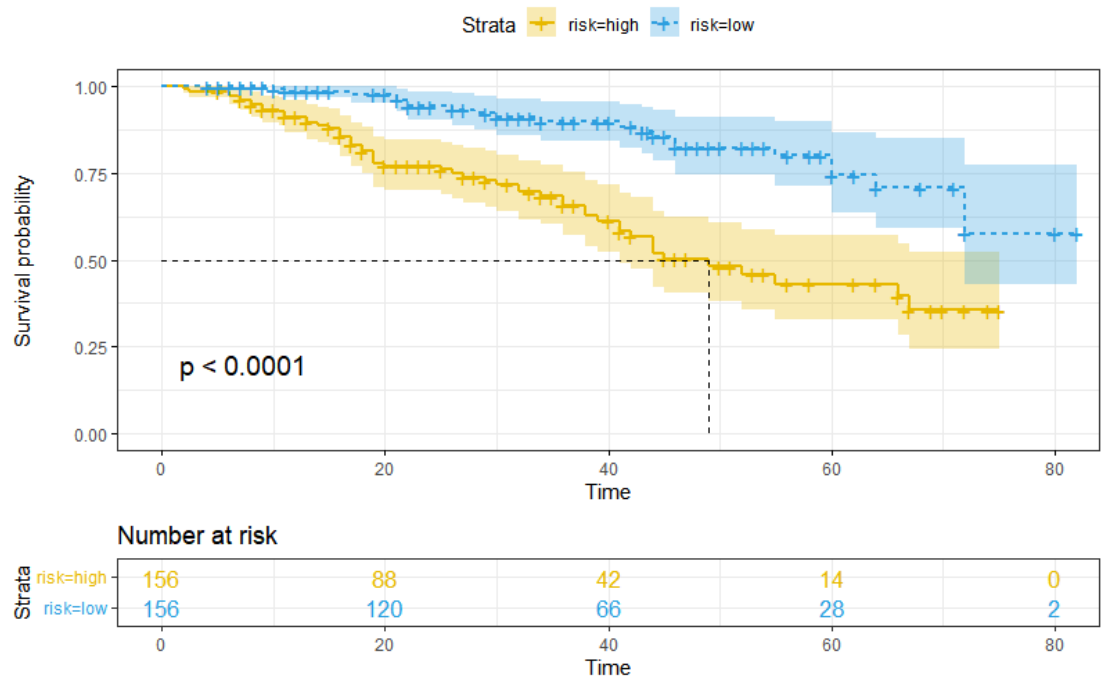

Supplement: Supplementary file 9 — Data S9. Supporting information. [file NICC-30-0-s007.pdf]

Supplement 10.Survival curves of high and low risk groups in the validation set

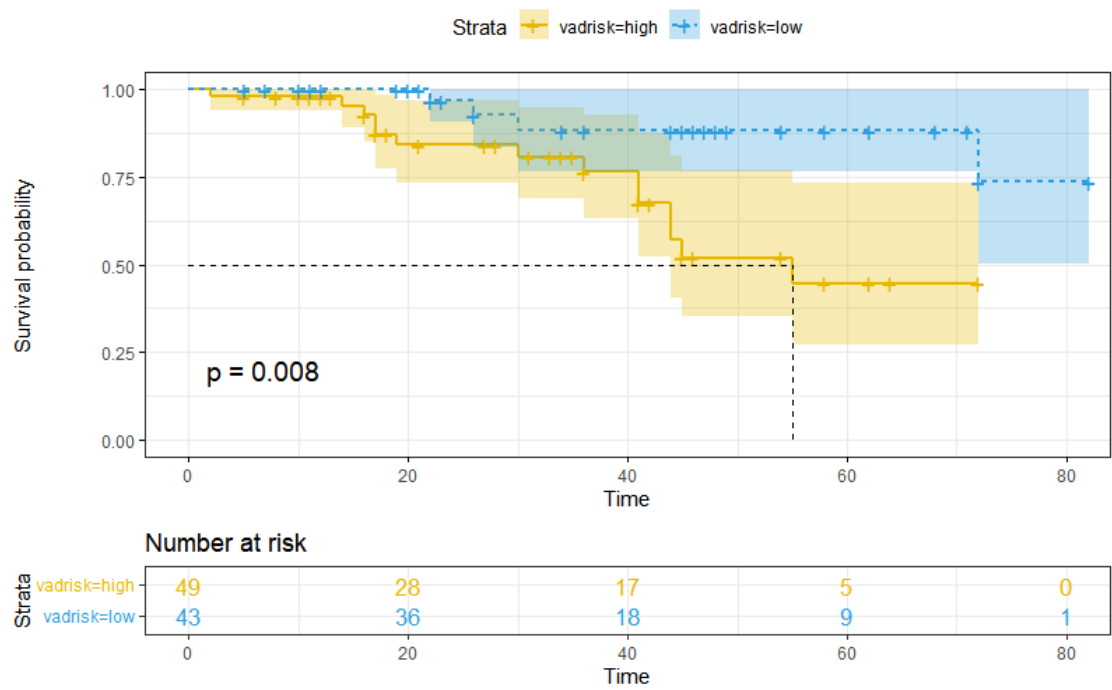

Supplement: Supplementary file 10 — Data S10. Supporting information. [file NICC-30-0-s003.pdf]
