# Supplementary material for: Development and validation of a nomogram for circuit lifespan of regional citrate anticoagulation‐continuous renal replacement therapy in intensive care patients with acute kidney injury
Source: Nurs Crit Care. 2024 Nov 7;30(4):e13196. doi: 10.1111/nicc.13196 (PMC12208818; doi:10.1111/nicc.13196)
Supplement: Supplementary file 2 — Data S2. Supporting information. [file NICC-30-0-s006.pdf]

Supplement 2. Lasso regression feature variable screening chart

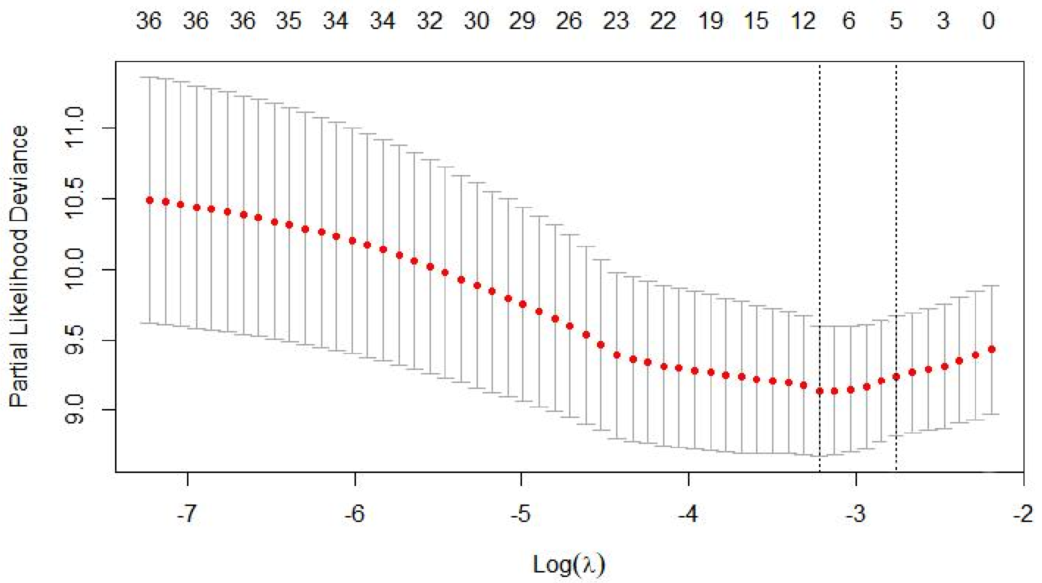

(a) Cross validation diagram

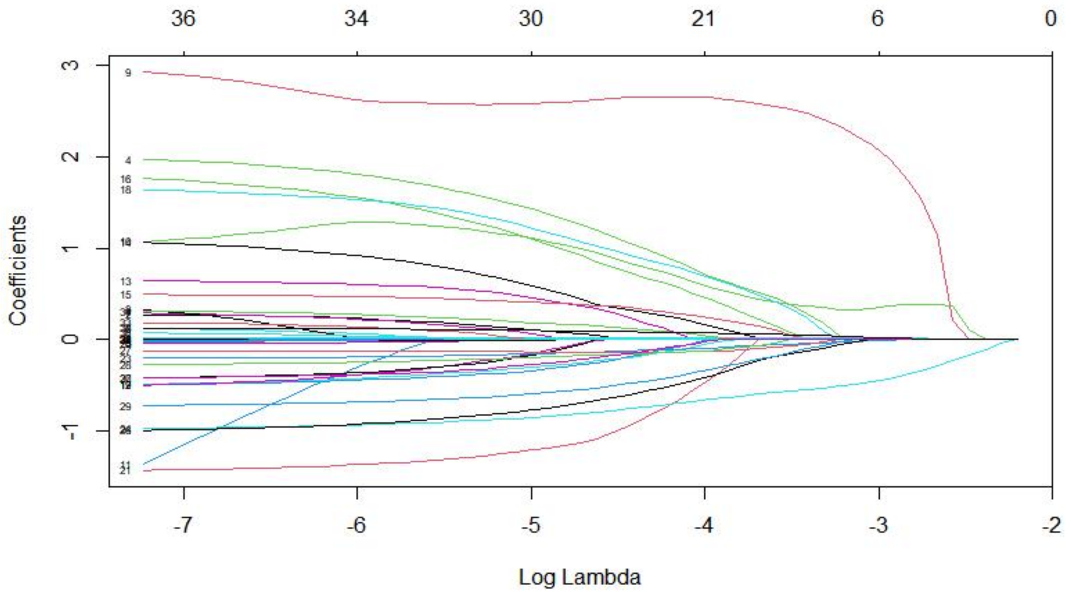

(b) Lasso coefficient path diagram for 24 variables

Abbreviations:Lasso regression analysis is a linear regression method that constrains model complexity by adding an L1 regularization term to the loss function. This approach aims to achieve feature selection by shrinking some regression coefficients to zero, thereby enhancing the interpretability and predictive accuracy of the model. The advantage of this method lies in its ability to prevent the issue of multicollinearity that may arise from traditional methods.
