# Supplementary material for: Development and validation of a nomogram for circuit lifespan of regional citrate anticoagulation‐continuous renal replacement therapy in intensive care patients with acute kidney injury
Source: Nurs Crit Care. 2024 Nov 7;30(4):e13196. doi: 10.1111/nicc.13196 (PMC12208818; doi:10.1111/nicc.13196)
Supplement: Supplementary file 5 — Data S5. Supporting information. [file NICC-30-0-s004.pdf]

Supplement 5. Decision curve of the training set for the RCA-CRRT circuit lifespan prediction model

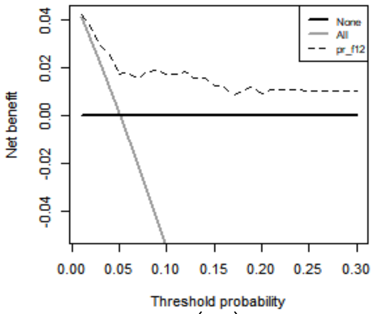

(12h)

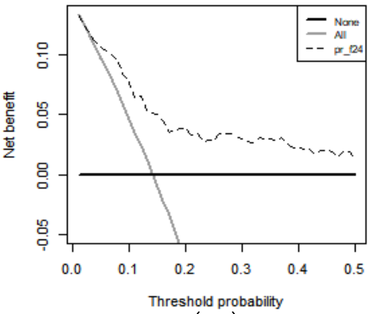

(24h)

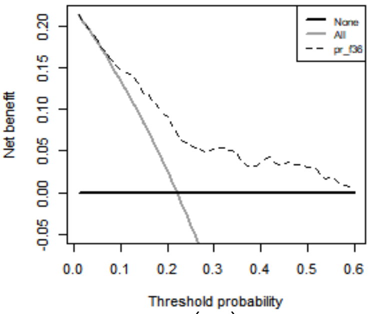

(36h)

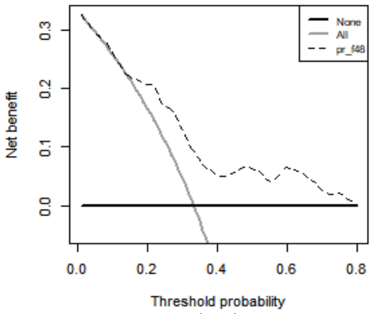

(48h)

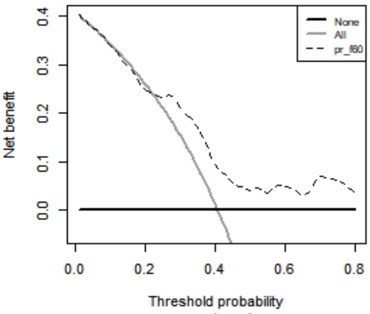

(60h)

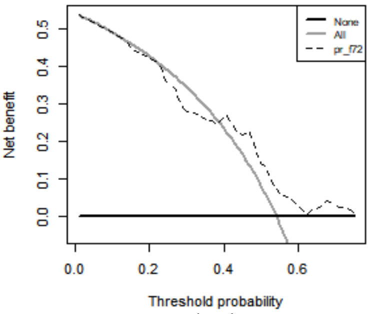

(72h)
