# Supplementary material for: Development and validation of a nomogram for circuit lifespan of regional citrate anticoagulation‐continuous renal replacement therapy in intensive care patients with acute kidney injury
Source: Nurs Crit Care. 2024 Nov 7;30(4):e13196. doi: 10.1111/nicc.13196 (PMC12208818; doi:10.1111/nicc.13196)

Supplement 8. Decision curve of the validation set for the RCA-CRRT circuit lifespan prediction model

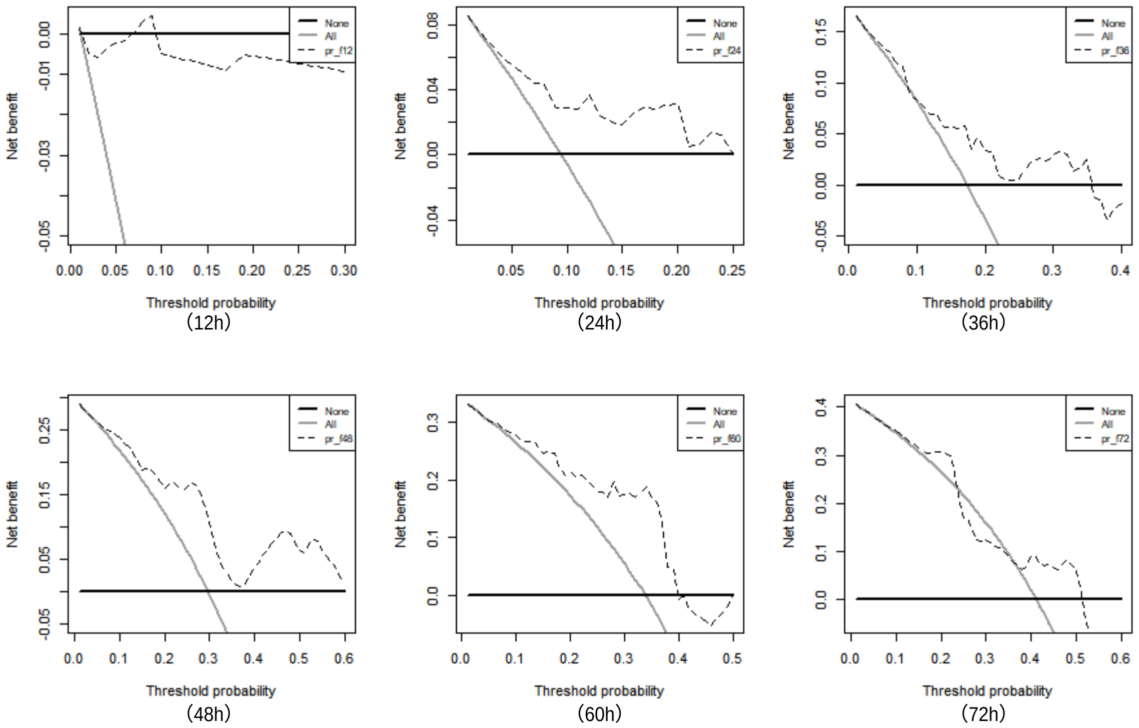

Supplement: Supplementary file 8 — Data S8. Supporting information. [file NICC-30-0-s001.pdf]
